# “It’s not you, it’s me”: identity disturbance as the main contributor to interpersonal problems in pathological narcissism

**DOI:** 10.1186/s40479-022-00209-6

**Published:** 2023-02-01

**Authors:** Marko Biberdzic, Junhao Tan, Nicholas J. S. Day

**Affiliations:** University of Wollongong, Illawarra Health and Medical Research Institute and School of Psychology, Wollongong, Australia

**Keywords:** Narcissism, Grandiosity, Vulnerability, Identity impairment, Interpersonal functioning

## Abstract

**Background:**

Core impairments in self and other functioning typify individuals with personality disorder. While interpersonal dysfunction is a known element of narcissistic disorders, empirical research investigating intrapersonal elements is lacking. The aim of this study was to investigate the internal representations of individuals with grandiose and vulnerable features, as manifested through their attachment styles, and the specific role of identity disturbance in explaining the relationship between pathological narcissism and maladaptive interpersonal functioning.

**Methods:**

A sample of 270 university students completed the Brief Pathological Narcissism Inventory (B-PNI), the Severity Indices of Personality Problems (SIPP), the Relationship Questionnaire (RQ), and the Inventory of Interpersonal Problems (IIP-32).

**Results:**

Both vulnerable and grandiose narcissism were positively associated with both fearful and preoccupied attachment, and negatively associated with secure attachment, whilst grandiose narcissism was also positively associated with dismissive attachment. Furthermore, unstable representations of self, poor self-reflective functioning, and low sense of purpose fully mediated the relationship between interpersonal problems and grandiose narcissism while partially mediating the relationship between interpersonal problems and vulnerable narcissism.

**Conclusions:**

Overall, our findings suggest that for individuals presenting with narcissistic features, capacity for adaptive interpersonal functioning is grounded by deficits in identity integration. Implications of these findings are discussed.

**Supplementary Information:**

The online version contains supplementary material available at 10.1186/s40479-022-00209-6.

Deficits in interpersonal functioning and disagreeable interpersonal interactions are a defining characteristic of pathological narcissism and narcissistic personality disorder [2, 42]. Individuals with narcissistic pathology are most often referred to treatment because of their impact on others, while often being oblivious to their own contribution to these interpersonal problems [63]. Deficits in self-functioning are also understood as a core indicator of general personality pathology, with unstable self-representations being linked with the development of the specific interpersonal difficulties commonly found in narcissistic individuals [45]. Nonetheless, despite existing theoretical and clinical contributions suggesting that distorted representations of self and others contribute to the problematic attitudes and interpersonal behaviours of narcissistic patients (e.g. [35, 43, 49, 61]), there is both limited and mixed empirical findings that support the specific association between pathological narcissism, identity disturbance, and interpersonal dysfunction. The current paper therefore aims to address this gap by investigating how internal relational representations underlying pathological narcissism – and unstable representations of self, more specifically – may explain the relationship between pathological narcissism and maladaptive interpersonal functioning.

## Pathological narcissism: a multifaceted construct

Pathological narcissism is commonly defined by significant self-regulatory deficits, a pervasive sense of entitlement, and maladaptive ways of defending against threats to a positive self-image [72]. It is also multifaceted in nature, with existing empirical literature consistently highlighting the existence of both grandiose and vulnerable phenotypes [28, 67, 84]. Grandiosity and vulnerability may each be expressed either overtly or covertly, with some individuals primarily displaying one or the other of these facets [66]. There is however growing consensus, that most narcissistic patients present with some degree of fluctuation between grandiose and vulnerable self-esteem states [23, 35, 66, 35, 73]. Although it has been noted that grandiose and vulnerable expressions of narcissism may represent different clinical manifestations of the disorder rather than discrete subtypes [44], the two phenotypic expressions of narcissistic pathology have shown both diverging and converging associations with a range of constructs, including interpersonal dysfunction (e.g., [21, 52, 74, 86]). Contemporary clinical conceptualizations of narcissistic pathology [44, 51, 62, 68, 72] thus suggest that grandiose and vulnerable expressions of narcissism are separable yet related expressions that manifest in distinct experiential and behavioural patterns (e.g. being self-assured and dominant vs. self-conscious and withdrawn when interacting with others), as reflected in recent empirical research [17]. Despite their seemingly contrasting presentations, both share (overtly or covertly expressed) attitudes of being special and entitled to special treatment [37, 51].

## Self and interpersonal functioning in pathological narcissism

Grandiose narcissism is typically characterized by attention seeking, arrogance, and self-enhancing and exaggerated self-worth. These individuals have been found to be interpersonally domineering, antagonistic and vindictive [14, 74, 75, 84]. They can also be socially charming despite being oblivious of others’ needs, interpersonally exploitative, and envious [55]. In contrast, the vulnerable type is characterized by defensiveness, withdrawal, emotional dysregulation, and hypersensitivity to others’ evaluations [38]. Gabbard [28] described these individuals as shy and “quietly grandiose,” with an “extreme sensitivity to slight,” which “leads to an assiduous avoidance of the spotlight.” Subsequent research has supported these clinical descriptions, with both types found to share a core of entitled and dismissive tendencies [30, 52].

It has been suggested that narcissistic individuals regulate their self-esteem through interpersonal dominance, denial of any form of reliance or dependency on others, and devaluation of others for not meeting expectations or for threatening their sense of superiority [13, 29, 64]. In this regard, Robbins and Dupont [70] describe a vicious cycle in which the problematic interpersonal behaviour of the narcissistic individual leads to a breakdown of interpersonal relations, followed by the accompanying reinforcement of these same problematic behaviours that are used to reinforce a fragile sense of self. These conflictual relationship patterns have been directly identified in empirical research, examining the moment-to-moment interactions between individuals with pathological narcissism and their partners or family members [18]. Similarly, Kernberg [44] argued that narcissistic individuals hold contradictory views of the self, which vacillate between the clinical expression of grandiose and vulnerable symptoms. Thus, the overtly narcissistic individual most frequently presents with grandiosity and exhibitionism. However, when the needs for admiration and idealized expectations of these individuals are not met, they become depressed, depleted, and feel painfully inferior. Pincus and colleagues have provided empirical backing to this understanding, supporting both the grandiose strivings toward self-enhancement as well as the expressions of vulnerability (e.g. social withdrawal and emotional dysregulation) following the painful disappointment of losses and failures [12, 67].

More recently, significant associations between narcissistic features and identity instability have also been established, with narcissism being linked with inflated but unstable representations of self [19, 22, 65, 71]. For example, Di Pierro and colleagues (2018) found identity instability to partially mediate the association between narcissistic traits and emotional empathy, highlighting the crucial role of internal representations of self when assessing severity of narcissistic functioning. This is in line with existing theoretical and clinical contributions that suggest that distorted internal representations of self and others contribute to the problematic attitudes and interpersonal behaviours of narcissistic patients. For example, representations of self as inferior and of others as potentially rejecting or domineering have been theorized to promote interpersonal withdrawal and avoidance [3, 43, 61], as reflected in recent empirical contributions [25, 85]. Similarly, a grandiose but unstable self-representation is believed to nurture exploitative interactions aimed at obtaining admiring responses from others, the lack of which may in turn foster rage and aggression [35, 49]. However, despite extensive support for the association between narcissistic pathology and interpersonal dysfunction as well as existing theoretical contributions, the specific mechanisms underlying this association are often overlooked in the empirical literature.

## Attachment and narcissistic pathology: internal working models of self and other

A useful framework and research paradigm for investigating the impact of internal representations of self and others on narcissistic interpersonal functioning is attachment theory. Attachment theory postulates that early life experiences have an enduring effect on personality development through the formation of cognitive-affective templates of self and other, termed internal working models [10]. These internal working models are believed to develop through repeated interactions with early caregivers and can be either positive or negative. For example, repeated benevolent experiences are reflected over time in the emergence of secure attachment patterns, characterized by positive models of self and others. On the other hand, inconsistent experiences of care or consistent rejection, neglect, or abuse, are thought to give rise to more problematic internal working models, in which others are viewed as emotionally unavailable or malevolent, and the self as unworthy of attention and care [59].

Internal working models have been operationalized through attachment styles, which reflect the relative security or insecurity of individuals’ attachment relationships [7, 54]. Insecure attachment has been associated with patterns of maladaptive personality functioning [11, 83], and has been identified as a risk factor for the development of narcissistic and borderline personality disorders [6, 57, 59]. Specifically, insecure attachment is typically classified into patterns of attachment anxiety and attachment avoidance, operationalized as either preoccupied/anxious (negative model of self; positive model of others), fearful/avoidant (negative models of self and others), or dismissive-avoidant (positive model of self; negative model of others) attachment styles. Attachment anxiety reflects fears of rejection and needs for reassurance in close relationships, with a tendency to feel distressed in response to perceived or anticipated separations. Attachment avoidance reflects discomfort with dependency and closeness, with tendencies toward detachment from relationships [11]. As a result, from an attachment perspective, personality dysfunction is understood to consist largely of the repeated activation of insecure attachment patterns, which reflect internal models of the self and others [58].

Despite their potential utility for improving our understanding of narcissistic pathology, relatively few studies have investigated the role of internal representations of self and others in the context of pathological narcissism. Moreover, the study of narcissism using an attachment paradigm has produced somewhat mixed findings. For example, Kaufman and Jauk [40] found no link between attachment and grandiose narcissism, while other research has associated grandiosity with avoidant and dismissive attachment [21, 87]. In contrast, vulnerable narcissism appears to be more consistently associated with insecure (anxious or avoidant) attachment, along with a negative working model of self [27, 40], or both [11, 21, 69, 80, 87].

## Current study

Considering the well-known deficits in interpersonal functioning in narcissistic pathology and the theorized role of unstable representations of self and others, the present study aimed to investigate whether grandiose and vulnerable features of pathological narcissism were associated with specific deficits in identity integration and attachment styles that contribute to interpersonal dysfunction, as well as to test the hypothesis that deficits in identity integration may indirectly explain the relationship between pathological narcissism and associated interpersonal problems. To our knowledge, no other research has investigated the role of identity disturbance as the underlying mechanism responsible for the association between narcissistic features and broader interpersonal dysfunction.

## Methods

### Participants and procedure

Two hundred seventy first-year university students aged between 18 to 25 years $(M=19.48, SD=1.732)$ took part in the current study. Participants were provided course credit for their participation. Amongst them, 33 were male $(12.22\%)$, 236 were female $(87.41\%)$, and one declared as non-binary $(0.37\%)$. All participants were English speaking. 103 participants $(38.15\%)$ reported having at least one previously diagnosed mental health condition and 147 participants $(54.44\%)$ reported having at least one immediate relative with a previously diagnosed mental health condition. Participation was voluntary and consent was obtained after reviewing the study’s information found in the participant information sheet. The study was approved by the University’s Human Research Ethics Committee.

### Measures

#### Brief-Pathological Narcissism Inventory (B-PNI)

The B-PNI [76] is a 28 item self-report questionnaire that assesses seven facets of pathological narcissism: Exploitativeness,Self-sacrificing self-enhancement; Grandiose fantasy; Contingent self-esteem; Hiding the self; Devaluing; and Entitlement rage. The B-PNI requires participants to rate the items based on a 5-point scale, ranging from 0 (*not at all like me*) to 4 (*very much like me*), with higher scores reflecting higher levels of narcissism. Within the current study, the B-PNI had high internal reliability $(\alpha =.91)$. Separately, both narcissistic grandiosity and vulnerability reported high reliabilities of $\alpha =.84$, and $\alpha =.88$ respectively.

#### Severity Indices of Personality Problems-118 (SIPP-118)

The SIPP-118 [81] is a dimensional self-report measure of the core components of (mal)adaptive personality functioning, and provides indices of the severity of personality pathology. In this study, only the Identity Integration domain was used, consisting of 36 items and covering five distinct facets: Self-respect (e.g. “I often feel that I am not as worthy as other people”); Stable self-image (e.g. “I am often confused about what kind of person I really am”); Self-reflexive functioning (e.g. “I am often not fully aware of my inner feelings”); Enjoyment (e.g. It is hard for me to really enjoy things); and Purposefulness (e.g. I often feel that my life is meaningless). SIPP-118 requires participants to rate the items based on a 4-point scale, ranging from 1 (*fully disagree*) to 4 (*fully agree*), with higher scores reflecting greater degrees of identity disturbance. Within the current study, the Identity Integration domain had high internal reliability $(\alpha =.93)$. Separately, self-respect, stable self-image, self-reflexive functioning, enjoyment, and purposefulness all reported high reliabilities of $\alpha =.88$, $\alpha =.81$, $\alpha =.78, \alpha =.85$, and $\alpha =.78$ respectively.

#### Relationship Questionnaire (RQ)

The RQ consists of four short paragraphs, each describing a prototypical attachment pattern as it applies to close relationships in adulthood. The four attachment styles (i.e. secure, fearful, preoccupied, and dismissing) are each represented by a single paragraph, which is rated “Strongly Disagree” (1) to “Strongly Agree” (7). Participants are then asked to rate their agreement with each prototype on a 7-point scale. The highest of the four attachment prototype ratings is then used to classify participants into an attachment category. The RQ is widely used and has demonstrated sound psychometric properties [5, 33].

#### Inventory of Interpersonal Problems (IIP-32)

The 32-item version of the IIP [4] was used to assess eight facets of interpersonal problems: Hard to be sociable, Hard to be assertive; Too aggressive; Too open; Too caring; Hard to be supportive; Hard to be involved; and Too dependent. IIP-32 requires participants to rate the items based on a 5-point scale, ranging from 0 (*not at all*) to 4 (*extremely*), with higher scores reflecting higher levels of interpersonal problems. In the current study, the IIP-32 had high internal reliability $(\alpha =.89)$.

### Statistical analyses

Preliminary and main analyses were conducted using Statistical Package for the Social Sciences (SPSS Version 25.0; [36]) software and Amos . Direct associations between levels of narcissism, attachment styles, identity disturbance, and interpersonal functioning were analysed using bivariate correlations. Mediation and moderated mediation analyses were then conducted to test for the mediating role of identity disturbance in the relationship between narcissism and interpersonal functioning using structural equation modelling (SEM).

## Results

### Descriptives, correlations and between-group differences

Preliminary analyses were conducted to determine the confounding effects of age, gender, and prior personal and family history of mental illness on the variables of interest. No significant effects of age were found through bivariate correlations. Meanwhile, significant differences between males and females were observed only on the grandiose narcissism variable, with male participants reporting significantly higher scores than female participants (Cohen’s *d* = 0.45). However, this difference was expected considering that males are known to exhibit higher levels of grandiosity compared to females [34]. Hence, gender was not controlled for in the main analyses. Finally, individuals with either prior personal or family history of mental illness scored significantly higher for interpersonal problems and identity integration. Thus, both forms of prior history of mental illness were controlled for in subsequent analyses.

Bivariate correlations were conducted to determine the direction and strength of the linear relationships between all of the main variables. As shown in Table 1, grandiose narcissism was positively correlated with fearful, preoccupied, and dismissive attachment, while vulnerable narcissism was positively correlated with fearful and preoccupied attachment and negatively correlated with secure attachment. With the exception of one facet of grandiose narcissism (exploitativeness), pathological narcissism was associated with deficits on all five domains of identity integration. Similarly, with the exception of exploitativeness, pathological narcissism as well as identity disturbance and attachment all correlated positively with interpersonal problems. Hence, all variables were included in the subsequent SEM analyses.

**Table 1:** Descriptive statistics for attachment, identity integration, narcissism, and interpersonal problems

Variable		1	2	3	4	5	6	7	8	9	10	11	12	13	14	15	16	*M* (SD)
Narcissism																		
1	Exploitativeness																	1.52 (.93)
2	Self-Sacrificing Self-Enhancement	.26**																2.06 (.82)
3	Grandiose Fantasy	.42**	.52**															2.08 (1.03)
4	Contingent Self Esteem	.25**	.46**	.50**														1.77 (.95)
5	Hiding the Self	.27**	.34**	.32**	.47**													2.01 (1.04)
6	Devaluing	.37**	.49**	.44**	.47**	.49**												1.32 (.93)
7	Entitlement Rage	.49**	.46**	.50**	.49**	.34**	.59**											1.31 (.83)
Identity Integration																		
8	Self-Respect	.00	.23**	.20**	.61**	.52**	.29**	.18**										2.18 (.69)
9	Stable Self- Image	.10	.23**	.22**	.49**	.44**	.36**	.27**	.68**									2.15 (.62)
10	Self-Reflexive Functioning	.05	.20**	.13*	.37**	.43**	.30**	.24**	.56**	.68**								2.23 (.61)
11	Enjoyment	.11	.16**	.17**	.41**	.45**	.36**	.25**	.58**	.57**	.55**							2.11 (.65)
12	Purposefulness	.07	.11	.22**	.32**	.39**	.27**	.20**	.53**	.63**	.53**	.50**						2.29 (.44)
Attachment																		
13	Secure	-.07	.03	-.11	-.19**	-.34**	-.16**	-.15*	-.26**	-.25**	-.22**	-.27**	-.18**					4.43 (1.86)
14	Fearful	.04	.06	.13*	.22**	.33**	.23**	.12*	.39**	.36**	.33**	.35**	.35**	-.35**				4.69 (1.76)
15	Preoccupied	-.06	.22**	.10	.27**	.10	.14*	.10	.27**	.20**	.13*	.16**	.17**	-.03	.14*			4.02 (1.75)
16	Dismissive	.14*	-.01	.11	-.16**	.07	.05	.02	-.10	-.01	-.00	.02	.10	-.07	.00	-.27**		4.11 (1.80)
Interpersonal Problems																		
17	Total Score	.09	.25**	.29**	.49**	.51**	.45**	.31**	.56**	.59**	.54**	.54**	.43**	-.39**	.37**	.21**	.03	1.39 (.54)

*N* = 270, * = *p* < .05, ** = *p* < .001

Moreover, One-Way Analyses of Variance (ANOVAs) were conducted to determine differences in means across the different attachment styles for each of the variables of interest. Results of the ANOVAs are outlined in Table 2. Overall, there were significant differences across the groups in terms of identity impairment, interpersonal functioning and vulnerable narcissism, but no significant differences relating to grandiose narcissism.

**Table 2:** Means, Standard Deviations, and One-Way Analyses of Variance for Narcissism, Identity Impairment, and Interpersonal Problems Across Attachment Styles

Variable	Secure (*n* = *82)*	Fearful/Avoidant (*n* = *103)*	Anxious/Preoccupied *(n* = *47)*	Dismissive *(n* = *38)*	$F(3,266)$	${\eta }^{2}$
	$M(SD)$	$M(SD)$	$M(SD)$	$M(SD)$		
GN	21.00 (8.61)^a^	23.39 (8.29)^a^	22.64 (9.05)^a^	24.34 (9.00)^a^	1.74	0.02
VN	19.77 (11.04)^a^	30.47 (10.93)^b^	25.91 (11.30)^bc^	24.97 (9.74)^ac^	14.80**	0.14
II	66.42 (13.55)^a^	87.87 (16.50)^b^	82.85 (14.28)^bc^	77.08 (17.52)^c^	30.66**	0.26
IIP	33.84 (17.37)^a^	52.49 (16.03)^bc^	45.45 (13.36)^b^	43.55 (13.48)^bd^	21.57**	0.20

*N* 270, Means with different superscripts are significantly different from each other, *GN* Grandiose narcissism, *VN* Vulnerable narcissism, *II* Identity impairment, *IIP* Interpersonal problems. ***p < .001

Post-hoc comparisons revealed that individuals with a secure attachment scored significantly lower on vulnerable narcissism, identity integration, and interpersonal problems when compared to their fearful/avoidant and anxious/preoccupied counterparts. These same individuals also scored significantly lower on identity integration and interpersonal problems when compared with those with a dismissive attachment. Similarly, those who endorsed a dismissive attachment style also scored significantly lower on vulnerable narcissism, identity integration, as well as interpersonal problems than those with a fearful/avoidant attachment.

### Structural equation modelling

**Fig. 1:**
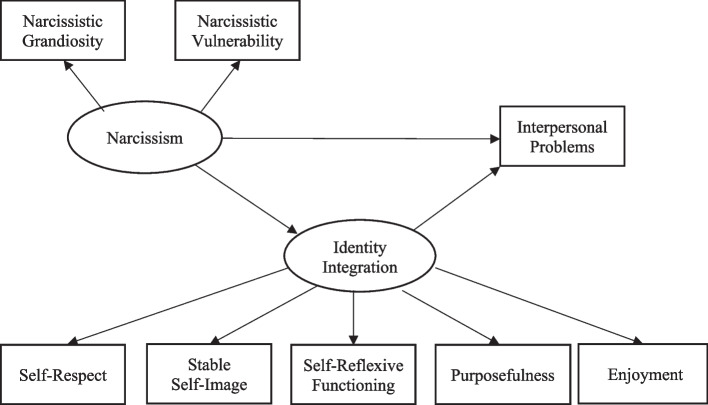
SEM illustrating the mediation model Note. Confounding variables were not included in the Figure. Standardized coefficients can be found in Table 2

SEM analyses were conducted to investigate the mediating effect of identity integration on the relationship between narcissism and interpersonal problems as illustrated in Fig. 1. In line with existing recommendations, mediation analyses were conducted via bootstrapping, allowing to estimate the confidence intervals of the indirect effects [77]. 5000 bootstrapped samples were used with a 95% confidence interval for each indirect effect. Results of SEM can be found on Table 3.

**Table 3:** Path Standardized Coefficients and Indirect Effects for Identity Integration as Mediator

	Path Coefficients							Indirect Effects	
	to Self-respect	to Stable Self-image	to Self-reflexive Functioning	to Purposefulness	to Enjoyment	to Interpersonal Problems		95% Confidence Interval	
							Estimate	Lower	Upper
Personal Mental Illness Hx	.189***	.119*	.008	.189***	.089	.074			
Family Mental Illness Hx	.066	.054	.155**	.025	.101	.071			
Narcissistic Grandiosity	-.264***	-.152*	-.216**	-.202**	-.123	-.059			
Narcissistic Vulnerability	.678***	.593***	.573***	.591**	.455***	.352***			
Self-respect						.081			
Stable Self-image						.206**			
Self-reflexive Functioning						.138*			
Purposefulness						.149*			
Enjoyment						-.022			
GN $\to$ SR $\to$ IIP							-.021	-.119	.023
**GN **$\to$** SSI **$\to$** IIP**							-.031*	-.137	-.016
**GN **$\to$** SRF **$\to$** IIP**							-.030*	-.133	-.011
**GN **$\to$** Pp **$\to$** IIP**							-.030**	-.129	-.019
GN $\to$ En $\to$ IIP							0.03	-.013	.045
VN $\to$ SR $\to$ IIP							.055	-.056	.205
**VN **$\to$** SSI **$\to$** IIP**							.122**	.090	.272
**VN **$\to$** SRF **$\to$** IIP**							.079*	.027	.208
**VN **$\to$** Pp **$\to$** IIP**							.088**	.045	.217
VN $\to$ En $\to$ IIP							-.010	-.073	-.047

*GN* Grandiose narcissism, *VN* Vulnerable narcissism, *SR* Self-respect, *SSI* Stable self-image, *SRF* Self-reflexive functioning, *En* Enjoyment, *Pp* Purposefulness, *IIP* Interpersonal problems

*N* = 270, * = *p* < .05*,* ** = *p* < .01, *** = *p* < .001

First, after accounting for the effects of prior personal and family history of mental illness, results showed personal history to have a significant relationship with self-respect ($\beta =.189, p<.001$), stable self-image ($\beta =.119, p<.05$), and purposefulness ($\beta =.189, p<.001$) while family history was significantly related to self-reflexive functioning ($\beta =.155, p<.01$). Neither forms of history were associated with interpersonal problems.

The mediation analysis showed both narcissistic grandiosity and vulnerability to have significant indirect effects on interpersonal problems through the various facets of identity integration, namely, stable self-image, self-reflexive functioning, and purposefulness. Goodness of fit indices indicated excellent fit for the overall model: ${\chi }^{2}\left(25\right)=36.70, p=.062$; CFI = 0.990; GFI = 0.974; NFI = 0.969; TLI = 0.982; RMSEA = 0.042; and SRMR = 0.038.

When looking at each of the two narcissism facets individually, results revealed a full mediating effect of stable self-image ($\beta =-.031, p<.05 :\mathrm{CI}=[-.137, -.016])$, self-reflexive functioning ($\beta =-.030, p<.05 :\mathrm{CI}=[-..133, -.011])$, and purposefulness ($\beta =-.030, p<.01 :\mathrm{CI}=[-.129, -.019])$ on the relationship between grandiose narcissism and interpersonal functioning. Similarly, vulnerable narcissism was shown to have significant indirect effects on interpersonal problems through stable self-image ($\beta =.122, p<.01 :\mathrm{CI}=[.090, .272])$, self-reflexive functioning ($\beta =.079, p<.05 :\mathrm{CI}=[.027, .208])$, and purposefulness ($\beta =.088, p<.01 :\mathrm{CI}=[.045, .217])$. The direct effects of narcissistic vulnerability on interpersonal problems remained however significant, suggesting a partial mediation.

As we were also interested in the potential role of representations of others (not just of self), the model was expanded to include the attachment variable. A moderating mediation analysis was therefore conducted with attachment moderating the association between identity integration and interpersonal problems (see Figure S1). Results however revealed a non-significant moderating effect while the mediating effect remained significant. Fit indices for the second model were also unsatisfactory: CMIN/$df$ = 3.46, GFI = 0.85, CFI = 0.79, NFI = 0.74, and RMSEA = 0.096.

## Discussion

The present study aimed to investigate the association between pathological narcissism and specific deficits in identity integration and attachment styles that contribute to interpersonal dysfunction, as well as to test the hypothesis that deficits in identity integration may indirectly explain the relationship between pathological narcissism and associated interpersonal problems.

Significant associations were found between deficits in identity integration, attachment styles, interpersonal problems, and both grandiose and vulnerable manifestations of pathological narcissism. Notably, different facets of grandiose narcissism were associated with all three types of insecure attachment styles, i.e. preoccupied, fearful, and dismissive. From an attachment perspective, this would suggest that grandiose narcissism involves both (split-off) positive and negative representations of self, and both (split-off) positive and dismissing attitudes toward others. Although grandiose narcissism has traditionally been associated with attachment avoidance and dismissal of the need for intimacy and closeness, our results also indicate an association with representations of self as inadequate, fearful, and sensitive to rejection. This suggests that a vulnerable experience of self is also a salient manifestation of narcissistic grandiosity. Despite mixed empirical results supporting these results [9, 18, 40, 42], this association is in line with existing theories of pathological narcissism, such as those advanced by Kernberg [43] and [48, 50], which argue that narcissistic grandiosity essentially covers and protects an implicitly negative self-representation and underlying feelings of inferiority and neediness. Subsequent theorists have also noted that narcissistic grandiosity is closely intertwined with external validation [66] despite fears and/or dismissal of dependency on others [15].

Similarly, vulnerable narcissism was mainly associated with both fearful and preoccupied attachment styles, indicating predominantly a negative model of self. Vulnerable narcissism was also negatively related to attachment styles denoting a positive model of self (i.e., dismissive and secure attachment). The association between vulnerability and both fearful and preoccupied attachment styles is in line with findings from Fossati and colleagues (2015) indicating that individuals high in narcissism are simultaneously anxious about maintaining attachments and reluctant to trust others. Moreover, individuals who were identified as having a preoccupied or fearful attachment style reported significantly higher scores on vulnerable narcissism as well as most severe deficits in identity integration and interpersonal functioning. These results are consistent with previous findings [21, 27, 42, 69, 78, 80, 87] and extend the importance of negative internal representations of self in relation to narcissistic functioning.

Moreover, results obtained from the subsequent mediation analysis lent additional support to the crucial role identity disturbance plays in explaining the relationship between pathological narcissism and overall interpersonal problems. In particular, deficits in identity integration partially mediated the relationship between vulnerable narcissism and interpersonal problems, while fully mediating the association between the latter and grandiose narcissism. Interestingly, the same facets of identity integration were found to play a significant role in these associations for both grandiose and vulnerable narcissism, namely Stable self-image, Self-reflexive functioning, and Purposefulness. These findings suggest that unstable self-representations, poor self-reflective functioning and lack of purpose in life are indirectly responsible for interpersonal difficulties reported in individuals presenting with narcissistic features. This in line with contemporary psychodynamic theories (e.g. [46], where identity diffusion (i.e. an unstable and unintegrated sense of self and of others) is understood as the root of borderline and narcissistic pathology, underlying many of the interpersonal problems that these individuals experience [45]. For example, identity diffusion is fundamentally characterized by problems with self–other boundaries, which may be expressed in fluctuations between extremely idealized and extremely devalued representations of self and others, leading to the typical interpersonal dynamics and difficulties reported in narcissistic patients. Similarly, identity diffusion has been linked to impairments in mentalizing, i.e. difficulties in reflective functioning [26]. Although studies on narcissism and reflective-functioning are sparse, existing studies have found individuals with NPD to have similar mentalizing deficits to those found in BPD (e.g. [20]. Our findings are in line with these results and suggest that deficits in self-reflective functioning may have an indirect impact on interpersonal problems in these individuals. This is also not surprising considering that the capacity to make sense of one’s internal experiences is key in our ability to navigate the social world [56].

As for the significant association between purposefulness and interpersonal problems, one explanation for this might be the potential shame and envy experienced by individuals with narcissistic features when facing the reality of failed long-term objectives. Items in the Purposefulness facet also reflected a lack of clear goals in life (e.g. ‘One of my problems is that I lack clear goals in my life’) as well as inconsistency (e.g. ‘My interests are changing all the time’), which may also be a by-product of an unstable and incoherent sense of self.

Of note, the associations between grandiose narcissism and both identity disturbance and interpersonal problems were negative in the SEM analysis – despite positive initial correlations between these variables. This may be explained by the presence of a suppression effect [82], which occurs when an independent variable contributes significantly to the tested model despite weak initial association with the dependant variable, i.e. despite being a weak predictor on its own. One interpretation of this negative association may be explained as a by-product of overall healthy personality functioning in participants. That is, by virtue of examining a non-clinical population, the participants in this study were relatively high functioning and as such the grandiosity expression under examination may be relatively adaptive or ‘healthy’. Such ‘healthy’ dimensions of narcissism have been historically theorised [47] as a normal or even essential part of psychological functioning. Such healthy or adaptive narcissism may include the capacity to maintain a positive self-image in the face or setbacks, or as reflecting a realistic sense of self-confidence and self-worth that promotes agency and autonomy – indeed, empirical evidence points to the presence of such a “high functioning/exhibitionistic narcissist” subtype [74]). However, as the narcissism measure utilised in this research captures an explicitly pathological expression, it is unlikely that the grandiosity captured in this study reflects such adaptive or ‘healthy’ narcissism. Instead, the negative association between grandiosity and psychopathology may instead reflect the image distorting defensive processes implicit in narcissistic disorders. That is, participants invested in a grandiose self-representation may under-report their degree of psychopathology in an effort to maintain their sense of superiority. Indeed, research has demonstrated that individuals with grandiose narcissism had less consistent and weaker self-reported indices of psychopathology, and instead were positively correlated with adaptive coping, life satisfaction, and image-distorting defense mechanisms [41]. That is, while pathological narcissism confers a defensive, self-protective element – this may in fact be a distortion of actual levels of functioning. Alternatively, such defensive processes may paradoxically actually improve functioning in certain domains (e.g., identity cohesion, occupational functioning), however the true ‘adaptiveness’ of this process is worth questioning [51] as the resulting narcissistic style often begets significant distress for those in the individual’s relational sphere [16, 60]. Supporting this notion of both image distortion and negative interpersonal impacts in narcissistic disorders, research has identified discrepancies between self and informant ratings of interpersonal functioning, with individuals with narcissistic features “putting a positive and self-enhancing spin on their personality while being described by peers as domineering, vindictive, and intrusive” [66], p. 435). Some variant of this process is a more likely explanation for the finding of lower reported psychopathology in our sample.

Interestingly, the ‘Self-respect’ facet of identity integration was found to be non-significant for both grandiose and vulnerable phenotypes. This may appear surprising at first considering that this scale contains items related to self-esteem (e.g. ‘I often feel that I am not as worthy as other people’) and sensitivity to criticism (e.g. ‘criticisms by others can make me feel very uncertain about myself’). However, closer examination of the subscale revealed that only negative views of self were examined (i.e. not fluctuations of self-esteem). It is therefore possible that the facet of ‘self-respect’ is more suited for the kind of low (and relatively stable) self-esteem issues found in individuals with depression, for example. Similarly, it is also interesting to note that the moderating mediation analysis that included attachment as a moderator of the association between identity integration and interpersonal problems was non-significant. Yet, the mediating effect of identity disturbance remained significant, suggesting that unstable representations of self indirectly impact interpersonal functioning in individuals with narcissistic features, regardless of their attachment style.

### Clinical implications

The findings presented in this study have important clinical implications. First, they suggest that achieving a sense of self-coherence for individuals with pathological narcissism may be a prerequisite for improving their interpersonal functioning. It is therefore not surprising that Transference Focused Psychotherapy (TFP) and Mentalization-Based Treatment (MBT) – two well-established treatments for personality disorders – both view identity disturbance as the focal point of treatment. Consistent with Kernberg’s theory, TFP attempts to achieve a coherent sense of self by helping the patient integrate split-off (polarized) representations of self and others, and by helping the patient learn to *reflect* on emotional states that were previously not understood (and were thus acted upon). In turn, MBT typically involves helping the patient mentalize about alternative views of self and others as a way of integrating extreme self and other representations, which ultimately leads to a stronger sense of self-coherence [24]. Second, our findings highlight the importance of considering specific interpersonal patterns and problems when working with individuals who present with narcissistic features. Although clinicians frequently recognize their patients’ interpersonal problems, they may overlook the potential narcissistic function of these behaviours [63]. Considering the interpersonal nature of psychotherapy, narcissistic interpersonal problems are inevitably brought directly into the treatment relationship (in the form of transference and countertransference [79]), and offer an opportunity to understand the underlying self-related difficulties. Finally, these results are consistent with the recent re-formulation of personality disorder in diagnostic systems (e.g., DSM-5, ICD-11, PDM-2) which propose a general level of personality impairment according to core self and other functional domains amongst more specific features. In this vein, our findings identify associations between such core impairments (operationalised as identity integration and interpersonal functioning with features that are narcissism specific as indexed by the B-PNI (e.g., grandiosity, vulnerability, entitlement).

### Limitations

While consistent with results from previous studies, findings from the current study should be considered in light of certain limitations. First, both expressions of narcissism were measured by the B-PNI. Although scores of both expressions of narcissism can be derived from the B-PNI’s subscales, some have questioned the PNI’s capacity to adequately capture grandiose narcissism [51]. Second, the current study was limited by its cross-sectional nature. Although the use of SEM in cross-sectional studies has been found to have sound psychometric value (see [8], measurements of narcissism were limited to trait-level conceptualizations [1]. Considering that grandiose and vulnerable manifestations of narcissism involve complex dynamic processes and are likely to wax and wane depending on social situations, this complexity might not have been captured by the current study’s methodological design. Third, all measures used in the current study were self-reported instruments and social desirability was not controlled for. Fourth, males were underrepresented in the current study as our sample was predominately composed of female participants (87%). Although the observed gender differences found in this study are consistent with the current literature, with males scoring exclusively higher on narcissistic grandiosity than females [34], recent empirical work on gender differences in pathological narcissism suggests gender disparities in both narcissistic expression and behaviour (for a review, see [31]. Considering that recent work has also highlighted that gender differences may arise in the endorsement of items pertaining to pathological narcissism (i.e., the items contained within instruments measuring pathological narcissism may have different meaning for the two genders, and more generally reflecting the male gender expression than that of females and feminine qualities, [32, 39], an equally-distributed sample across both genders as well as the inclusion of different measures of pathological narcissism would allow for a more statistically sound and clinically relevant investigation of potential gender differences. This was however beyond the scope of the current study. Finally, in extension to the previous limitation, while the current sample was drawn from a population of undergraduates it is important to note that the narcissism measure used for analysis has an explicit focus on psychopathology. That is, this study was not exploring so called ‘adaptive’ or ‘normal’ narcissism, but instead identified associations between variations in *pathological* narcissism and underlying issues of identity integration. It is possible that alternate narcissism constructs may be less related to such features, but as such these findings provide a more clinically relevant perspective, and future studies should consider replicating the current findings within a clinical population to support their generalizability.

## Conclusion

Overall, our findings suggest that for individuals presenting with narcissistic features, capacity for self and interpersonal functioning are related constructs. That is, while grandiosity provides a façade of stable self-functioning and vulnerability presents a more desolate and fragile self-experience, both are marked by impaired interpersonal functioning as related to a broad underlying disorder of self-identity integration. In this way, grandiose and vulnerable functioning are linked, reflecting a common core of impairment, and perhaps best viewed as reflecting two sides of the same coin [53]. This view is consistent with contemporary diagnostic systems of personality, which account for core impairments in functioning but appreciate the diversity of presentation in personality ‘traits’ or ‘styles’. Ultimately, these findings stress the importance of accounting for intrapersonal factors in the assessment and treatment of narcissism, as opposed to or in conjunction with the perhaps more observable and apparent features relating to the discordant style of interacting with others.

## Supplementary Information

**Additional file 1:** **Figure S1.** SEM illustrating the moderated-mediation model.

## Data Availability

The datasets generated and/or analysed during the current study do not have clearance to be made publicly available but the analysis script can be made obtained from the corresponding author on reasonable request.

## References

[1] Ackerman RA, Donnellan MB, Wright AGC (2019). Current conceptualization of narcissism. Curr Opin Psychiatry.

[2] American Psychiatric Association. Diagnostic and statistical manual of mental disorders (5th ed.). Arlington: 2013. 10.1176/appi.books.9780890425596.

[3] Bacal HA, Newman KM, Newman K. Theories of object relations: Bridges to self psychology. Columbia University Press; 1990.

[4] Barkham M, Hardy GE, Startup M (1996). The IIP-32: A short version of the inventory of interpersonal problems. Br J Clin Psychol.

[5] Bartolomew K, Horowitz LM (1991). Attachment styles among young adults: A test of a four-category model. J Pers Soc Psychol.

[6] Bennett CS (2006). Attachment theory and research applied to the conceptualization and treatment of pathological narcissism. Clin Soc Work J.

[7] Blatt SJ, Auerbach JS, Levy KN (1997). Mental representations in personality development, psychopathology, and the therapeutic process. Rev Gen Psychol.

[8] Bollen KA, Pearl J. Eight myths about causality and structural equation models. In: Handbook of causal analysis for social research. Springer; 2013. p. 301–328.

[9] Bosson JK, Lakey CE, Campbell WK, Zeigler-Hill V, Jordan CH, Kernis MH (2008). Untangling the links between narcissism and self-esteem: A theoretical and empirical review. Soc Pers Psychol Compass.

[10] Bowlby J (1988). Developmental psychiatry comes of age. Am J Psychiatry..

[11] Brennan KA, Shaver PR (1998). Attachment styles and personality disorders: Their connections to each other and to parental divorce, parental death, and perceptions of parental caregiving. J Pers.

[12] Cain NM, Pincus AL, Ansell EB (2008). Narcissism at the crossroads: Phenotypic description of pathological narcissism across clinical theory, social/personality psychology, and psychiatric diagnosis. Clin Psychol Rev.

[13] Campbell WK, Baumeister RF. Narcissistic Personality Disorder. In: Fisher JE, O'Donohue WT, editors. Practitioner's guide to evidence-based psychotherapy. Springer Science + Business Media. 2006. p. 423–431. 10.1007/978-0-387-28370-8_42.

[14] Cheek J, Kealy D, Joyce AS, Ogrodniczuk JS (2018). Interpersonal problems associated with narcissism among psychiatric outpatients: A replication study. Arch Psychiatry Psychother.

[15] Cooper J, Maxwell N (1995). Narcissistic Wounds: Clinical Perspectives.

[16] Day NJS, Bourke ME, Townsend ML, Grenyer BFS. Pathological narcissism: A study of burden on partners and family. J Personal Disord. 2019:33. 10.1521/pedi_2019_33_413 10.1521/pedi_2019_33_413 30730784

[17] Day NJ, Townsend ML, Grenyer BF (2020). Living with pathological narcissism: a qualitative study. Borderline Personality Disorder and Emotion Dysregulation.

[18] Day NJ, Townsend ML, Grenyer BF (2022). Pathological narcissism: An analysis of interpersonal dysfunction within intimate relationships. Personal Ment Health.

[19] Di Pierro R, Di Sarno M, Preti E, Di Mattei VE, Madeddu F (2018). The role of identity instability in the relationship between narcissism and emotional empathy. Psychoanal Psychol.

[20] Diamond D, Levy KN, Clarkin JF, Fischer-Kern M, Cain NM, Doering S, Buchheim A (2014). Attachment and mentalization in female patients with comorbid narcissistic and borderline personality disorder. Personal Disord Theory Res Treat.

[21] Dickinson KA, Pincus AL (2003). Interpersonal analysis of grandiose and vulnerable narcissism. J Pers Disord.

[22] Dimaggio G, Procacci M, Nicolò G, Popolo R, Semerari A, Carcione A, Lysaker PH (2007). Poor metacognition in narcissistic and avoidant personality disorders: four psychotherapy patients analysed using the Metacognition Assessment Scale. Clin Psychol Psychother.

[23] Dimaggio G, Semerari A, Falcone M, Nicolo G, Carcione A, Procacci M (2002). Metacognition, states of mind, cognitive biases, and interpersonal cycles: Proposal for an integrated narcissism model. J Psychother Integr.

[24] Drozek RP, Unruh BT (2020). Mentalization-Based Treatment for Pathological Narcissism. J Pers Disord.

[25] Edershile EA, Wright AG (2021). Fluctuations in grandiose and vulnerable narcissistic states: A momentary perspective. J Pers Soc Psychol.

[26] Fonagy P, Gergely G, Jurist EL, Target M. Affect regulation, mentalization, and the development of the self. Routledge; 2018.

[27] Fossati A, Feeney J, Pincus A, Borroni S, Maffei C (2015). The structure of pathological narcissism and its relationships with adult attachment styles: A study of Italian nonclinical and clinical adult participants. Psychoanal Psychol.

[28] Gabbard GO (1989). Two subtypes of narcissistic personality disorder. Bull Menninger Clin.

[29] Gabbard GO. Transference and countertransference in the treatment of narcissistic patients. In: Ronningstam EF, editors. Disorders of narcissism: Diagnostic, clinical, and empirical implications. American Psychiatric Association. 1998. p. 125–145.

[30] Glover N, Miller JD, Lynam DR, Crego C, Widiger TA (2012). The five-factor narcissism inventory: A five-factor measure of narcissistic personality traits. J Pers Assess.

[31] Green A, MacLean R, Charles K (2020). Unmasking gender differences in narcissism within intimate partner violence. Personality Individ Differ.

[32] Green A, MacLean R, Charles K (2022). Female narcissism: Assessment, aetiology, and behavioural manifestations. Psychol Rep.

[33] Griffin DW, Bartholomew K (1994). Models of the self and other: Fundamental dimensions underlying measures of adult attachment. J Pers Soc Psychol.

[34] Grijalva E, Newman DA, Tay L, Donnellan MB, Harms PD, Robins RW, Yan T (2015). Gender differences in narcissism: A meta-analytic review. Psychol Bull.

[35] Horowitz M (2009). Clinical phenomenology of narcissistic pathology. Psychiatr Ann..

[36] IBM Corp. Released 2017. IBM SPSS Statistics for Windows, Version 25.0. Armonk, NY: IBM Corp.

[37] Jauk E, Kanske P. Can neuroscience help to understand narcissism? A systematic review of an emerging field. Personal Neurosci. 2021;4:e3,1–29. 10.1017/pen.2021.1. 10.1017/pen.2021.1 PMC8170532 34124536

[38] Jauk E, Weigel E, Lehmann K, Benedek M, Neubauer AC (2017). The relationship between grandiose and vulnerable (hypersensitive) narcissism. Front Psychol.

[39] Jonason PK, Davis MD (2018). A gender role view of the dark triad traits. Personality Individ Differ.

[40] Kaufman SB, Jauk E. Healthy selfishness and pathological altruism: Measuring two paradoxical forms of selfishness. Front Psychol. 2020;1006. 10.3389/fpsyg.2020.01006. 10.3389/fpsyg.2020.01006 PMC7265883 32528378

[41] Kaufman SB, Weiss B, Miller JD, Campbell WK (2018). Clinical correlates of vulnerable and grandiose narcissism: a personality perspective. J Pers Disord.

[42] Kealy D, Ogrodniczuk JS, Joyce AS, Steinberg PI, Piper WE (2015). Narcissism and relational representations among psychiatric outpatients. J Pers Disord.

[43] Kernberg OF (1984). Severe Personality Disorders: Psychotherapeutic Strategies.

[44] Kernberg OF, Ronningstam EF (1998). Pathological narcissism and narcissistic personality disorder: Theoretical background and diagnostic classification. Disorders of narcissism: Diagnostic, clinical, and empirical implications.

[45] Kernberg OF. Narcissistic personality disorder. In: Clarkin JF, Fonagy P, Gabbard GO, editors. Psychodynamic psychotherapy for personality disorders: A clinical handbook. 2010. p. 257–287.

[46] Kernberg OF, Caligor E (2005). A psychoanalytic theory of personality disorders. Major theories of personality disorder.

[47] Kohut H (1966). Forms and transformations of narcissism. J Am Psychoanal Assoc.

[48] Kohut H (1968). The psychoanalytic treatment of narcissistic personality disorders: Outline of a systematic approach. Psychoanal Study Child.

[49] Kohut H (1972). Thoughts on narcissism and narcissistic rage. Psychoanal Study Child.

[50] Kohut H, Wolf ES (1978). The disorders of the self and their treatment: An outline. International Journal of Psycho-Analysis.

[51] Krizan Z, Herlache AD (2018). The narcissism spectrum model: A synthetic view of narcissistic personality. Pers Soc Psychol Rev.

[52] Krizan Z, Johar O (2012). Envy divides the two faces of narcissism. J Pers.

[53] Levy KN (2012). Subtypes, dimensions, levels, and mental states in narcissism and narcissistic personality disorder. J Clin Psychol.

[54] Levy KN, Blatt SJ, Shaver PR (1998). Attachment styles and parental representations. J Pers Soc Psychol.

[55] Levy KN, Chauhan P, Clarkin JF, Wasserman RH, Reynoso JS. Narcissistic pathology: Empirical approaches. Psychiatr Ann. 2009;39(4):203–13. 10.3928/00485713-20090401-03.

[56] Luyten P, Fonagy P, Lowyck B, Vermote R, Bateman AW, Fonagy P (2012). Assessment of mentalization. Handbook of mentalizing in mental health practice.

[57] Maxwell K, Huprich S (2014). Retrospective reports of attachment disruptions, parental abuse and neglect mediate the relationship between pathological narcissism and self-esteem. Personal Ment Health.

[58] Meyer B, Pilkonis PA. An Attachment Model of Personality Disorders. In: Lenzenweger MF, Clarkin JF, editors. Major theories of personality disorder. Guilford Press; 2005. p. 231–281.

[59] Meyer B, Pilkonis PA, Campbell WK, Miller JD (2011). Attachment theory and narcissistic personality disorder. The handbook of narcissism and narcissistic personality disorder: Theoretical approaches, empirical findings, and treatments.

[60] Miller JD, Campbell WK, Pilkonis PA (2007). Narcissistic personality disorder: relations with distress and functional impairment. Compr Psychiatry.

[61] Modell AH (1975). A narcissistic defence against affects and the illusion of self-sufficiency. International Journal of Psycho-Analysis.

[62] Morf CC, Torchetti L, Schurch E, Campbell WK, Miller JD (2011). Narcissism from the perspective of the dynamic self-regulatory processing model. The handbook of narcissism and narcissistic personality disorder: Theoretical approaches, empirical findings, and treatments.

[63] Ogrodniczuk JS, Kealy D, Ogrodniczuk JS (2013). Interpersonal problems of narcissistic patients. Understanding and treating pathological narcissism.

[64] Ogrodniczuk JS, Piper WE, Joyce AS, Steinberg PI, Duggal S (2009). Interpersonal problems associated with narcissism among psychiatric outpatients. J Psychiatry Res..

[65] Pincus AL, Ansell EB, Pimentel CA, Cain NM, Wright AGC, Levy KN (2009). Initial construction and validation of the pathological narcissism inventory. Psychol Assess.

[66] Pincus AL, Lukowitsky MR (2010). Pathological narcissism and narcissistic personality disorder. Annu Rev Clin Psychol.

[67] Pincus AL, Roche MJ. Narcissistic Grandiosity and Narcissistic Vulnerability. In: Campbell WK, Miller JD, editors. The Handbook of Narcissism and Narcissistic Personality Disorder. 2011. p. 31–40. 10.1002/9781118093108.ch4.

[68] Pincus AL, Roche MJ, Good EW, Blaney PH, Krueger RF, Millon T (2015). Narcissistic personality disorder and pathological narcissism. Oxford textbook of psychopathology.

[69] Reis S, Huxley E, Eng YFB, Grenyer BF (2021). Pathological Narcissism and Emotional Responses to Rejection: The Impact of Adult Attachment. Front Psychol.

[70] Robbins SB, Dupont P (1992). Narcissistic needs of the self and perceptions of interpersonal behavior. J Couns Psychol.

[71] Roche MJ, Pincus AL, Lukowitsky MR, Ménard KS, Conroy DE (2013). An integrative approach to the assessment of narcissism. J Pers Assess.

[72] Ronningstam E (2005). Identifying and understanding the narcissistic personality.

[73] Ronningstam E (2011). Narcissistic personality disorder in DSM-V–in support of retaining a significant diagnosis. J Personal Disord..

[74] Russ E, Shedler J, Bradley R, Westen D (2008). Refining the construct of narcissistic personality disorder: Diagnostic criteria and subtypes. Am J Psychiatry.

[75] Samuel DB, Widiger TA (2008). Convergence of narcissism measures from the perspective of general personality functioning. Assessment.

[76] Schoenleber M, Roche MJ, Wetzel E, Pincus AL, Roberts BW (2015). Development of a brief version of the pathological narcissism inventory. Psychol Assess.

[77] Shrout PE, Bolger N (2002). Mediation in experimental and nonexperimental studies: new procedures and recommendations. Psychol Methods.

[78] Smolewska K, Dion K (2005). Narcissism and adult attachment: A multivariate approach. Self and Identity.

[79] Tanzilli A, Muzi L, Ronningstam E, Lingiardi V (2017). Countertransference when working with narcissistic personality disorder: An empirical investigation. Psychotherapy.

[80] van Schie CC, Jarman HL, Reis S, Grenyer BF (2021). Narcissistic traits in young people and how experiencing shame relates to current attachment challenges. BMC Psychiatry.

[81] Verheul R, Andrea H, Berghout CC, Dolan C, Busschbach JJ, van der Kroft PJ, Fonagy P (2008). Severity Indices of Personality Problems (SIPP-118): Development, factor structure, reliability, and validity. Psychol Assess.

[82] Watson D, Clark LA, Chmielewski M, Kotov R (2013). The value of suppressor effects in explicating the construct validity of symptom measures. Psychol Assess.

[83] Westen D, Nakash O, Thomas C, Bradley R (2006). Clinical assessment of attachment patterns and personality disorder in adolescents and adults. J Consult Clin Psychol.

[84] Wink P (1991). Two faces of narcissism. J Pers Soc Psychol.

[85] Wright AG, Stepp SD, Scott LN, Hallquist MN, Beeney JE, Lazarus SA, Pilkonis PA (2017). The effect of pathological narcissism on interpersonal and affective processes in social interactions. J Abnorm Psychol.

[86] Zeigler-Hill V, Besser A (2013). A glimpse behind the mask: Facets of narcissism and feelings of self-worth. J Pers Assess.

[87] Zhang Q, Zhang L, Li C (2017). Attachment, perceived parental trust and grandiose narcissism: Moderated mediation models. Personality Individ Differ.

