# Correction: “It’s not you, it’s me”: identity disturbance as the main contributor to interpersonal problems in pathological narcissism

**DOI:** 10.1186/s40479-023-00214-3

**Published:** 2023-03-03

**Authors:** Marko Biberdzic, Junhao Tan, Nicholas J. S. Day

**Affiliations:** University of Wollongong, Illawarra Health and Medical Research Institute and School of Psychology, Wollongong, Australia

**Correction: Borderline Personal Disord Emot Dysregul 10, 3 (2023)**

**https://doi.org/10.1186/s40479-022-00209-6**

Following publication of the original article [1], we have been notified that the authors’ first and last names were not tagged correctly. Correct tagging should be as per below:

given name – Marko

family name - Biberdzic

given name - Junhao

family name - Tan

given name - Nicholas J.S.

family name - Day
