# Supplementary material for: “It’s not you, it’s me”: identity disturbance as the main contributor to interpersonal problems in pathological narcissism
Source: Borderline Personal Disord Emot Dysregul. 2023 Feb 1;10:3. doi: 10.1186/s40479-022-00209-6 (PMC9890803; doi:10.1186/s40479-022-00209-6)
Supplement: Supplementary file 1 — Additional file 1: Figure S1. SEM illustrating the moderated-mediation model. [file 40479_2022_209_MOESM1_ESM.docx]

**Figure S1**

*SEM illustrating the moderated-mediation model*

Self-Reflexive

Functioning

Enjoyment

Purposefulness

Stable

Self-Image

Self-Respect

Interpersonal Problems

Narcissistic Grandiosity

Narcissistic Vulnerability

Attachment Styles
